# Ten simple rules for successfully carrying out funded research projects

**DOI:** 10.1371/journal.pcbi.1012431

**Published:** 2024-09-19

**Authors:** Diego A. Forero, Walter H. Curioso, Wei Wang

**Affiliations:** 1 School of Heath and Sport Sciences, Fundación Universitaria del Área Andina, Bogotá, Colombia; 2 Vicerrectorado de Investigación, Universidad Continental, Lima, Peru; 3 Clinical Research Centre, The First Affiliated Hospital of Shantou University Medical College, Shantou, China; 4 School of Public Health, Shandong First Medical University & Shandong Academy of Medical Sciences, Jinan, Shandong, China; 5 Beijing Key Laboratory of Clinical Epidemiology, Capital Medical University, Beijing, China; 6 Centre for Precision Health, Edith Cowan University, Perth, Australia; Carnegie Mellon University, UNITED STATES OF AMERICA

## Introduction

Receiving research funding, from external or internal sources, is one of the most important and challenging tasks for investigators around the world [1,2]. There are many prestigious research funding organizations, such as the National Institutes of Health of the US (NIH), the National Health Service of the UK (NHS), the National Science Foundation of the US (NSF), the European Commission (EC), the National Natural Science Foundation of China (NSFC), and the Japan Society for the Promotion of Science (JSPS), among many others. Although several scientific articles have provided important advice on how to write adequate research proposals and how to present them to be funded [3–5], how to become a principal investigator [6], and/or how to establish a laboratory [7], there is still a scarcity of articles addressing how to carry out research projects successfully and in an ethical way after the proposal has been granted.

Obtaining funding is usually the beginning of the research cycle [2] and an adequate implementation of the scientific activities, as proposed in the grant application, is of paramount importance for the generation of new knowledge, the preservation of scientific collaborations, and the academic advancement of the researchers [1].

**Fig 1:**
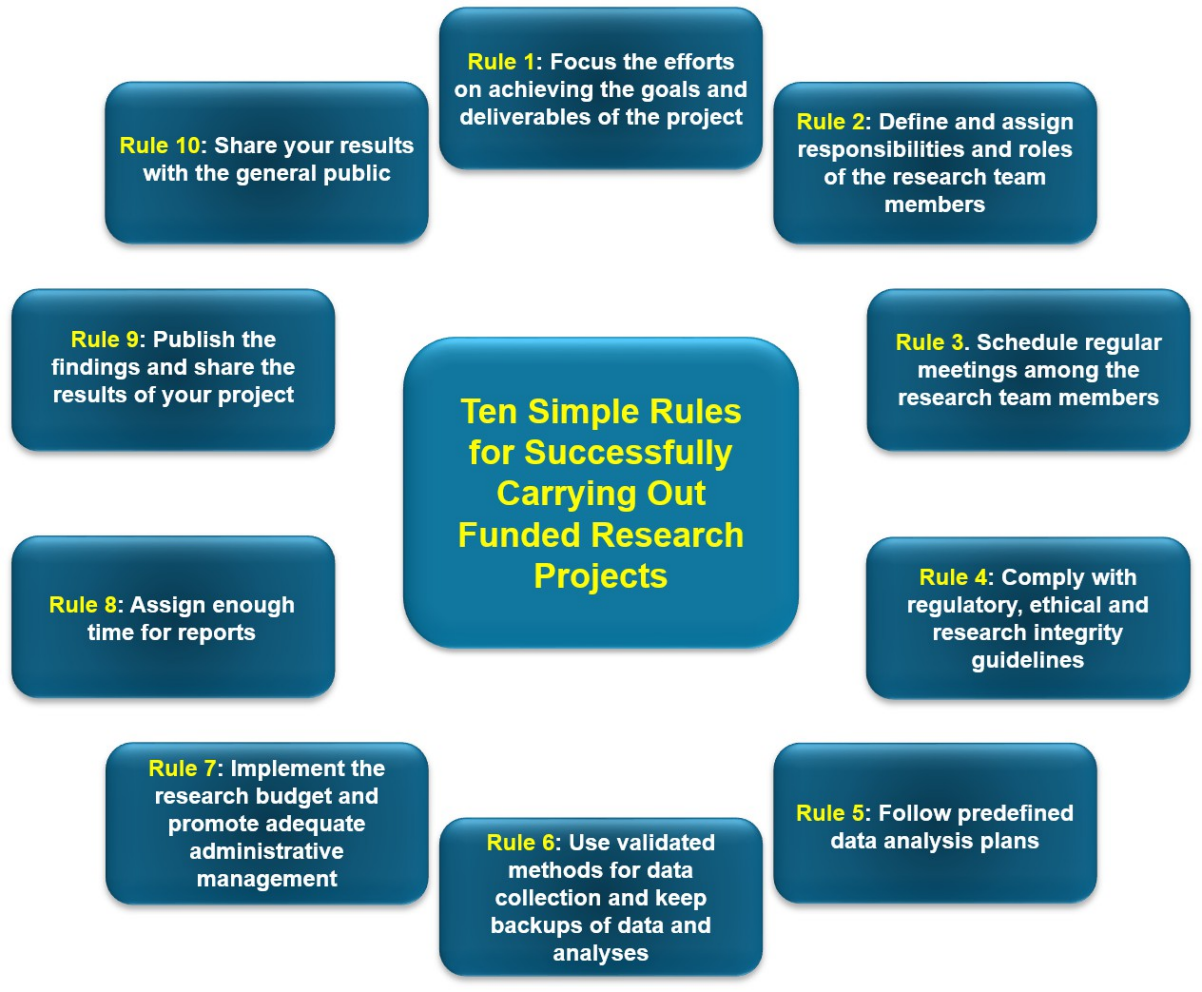
An overview of the Ten Simple Rules.

In these Ten Simple Rules, we provide valuable recommendations for successfully carrying out funded research projects, from our perspective and experience as both researchers and peer reviewers. These Ten Simple Rules are focused on activities carried out after a grant is awarded and they will be particularly useful for junior researchers globally. Regarding the presentation order of these Ten Simple Rules, some of them involve activities that are sequential (such as Rules 5, 6, and 9) and others comprise actions in parallel (such as Rules 3, 4, and 7). A graphical overview of the proposed Ten Simple Rules is presented in Fig 1.

## Rule 1: Focus the efforts on achieving the goals and deliverables of the project

Usually, funding bodies generate a contract, or a similar document, with the defined deliverables of the research project, such as peer-reviewed publications, presentations, patent applications, training of students, and public outreach activities, among others. In many cases, the contract is sent to administrative offices in the institution of the principal investigator and, depending on the funder, the expected deliverables are previously defined in the call for applications. The contract, or a similar document, will be a key guide from the start of the project, as the timeline, and budget, of a proposal is focused on the generation of those deliverables and the achievement of the proposed research goals; considering those deliverables from an early stage of the project will be important for an adequate and successful execution. Commonly, the timeline of a research project is presented in a Gantt chart, describing the main activities and the corresponding months or weeks projected for their execution [8].

## Rule 2: Define and assign responsibilities and roles of the research team members

Commonly, the grant application involves researchers with specific roles, such as the Principal Investigator (PI, the person leading the project) and the coinvestigators, who are proposed as responsible for certain activities, such as evaluation of patients or animals or statistical analysis of data, based on their academic profiles and research experience. In large projects, there is a possibility of hiring a Project Manager (PM), who is responsible for coordinating multiple scientific and administrative tasks [9]. Each member of a research team should have clear responsibilities, in order to complete the expected tasks in the predefined timeline and to avoid conflicts between them; it is of particular relevance for multicenter studies or for projects with challenging topics or methods. Other roles involve external advisors, administrative staff or students, each with a specific participation in the research project. It is advisable to plan for contingencies related to team members, such as having standard procedures for handling transitions associated with the arrival or departure of staff. Details of authorship should be reviewed in advance, following international recommendations and taking into account the main principles of research integrity [10].

## Rule 3: Schedule regular meetings among the research team members

Adequate communication among members of a research team, and between sub teams, is a key aspect in a research project. Regular meetings facilitate the periodic presentation of advances, in addition to providing a platform for discussion and documentation of findings and of potential challenges. In addition, consultations with administrative personnel from institutional offices (such as those related to budget or research oversight) are also important. Meetings should have a well-structured agenda about key issues and their frequency should be balanced, in order to avoid having too many sessions and wasting the valuable time of researchers.

An early identification and management of issues, such as difficult situations in communication between the research team or failures in experiments, may avoid occurrence of larger problems in the future. In the context of multi-institutional and international collaborations (which have particular challenges in terms of needing further definitions of roles and responsibilities), online systems for videoconferencing are time efficient and cost effective approaches for project meetings, in addition to in-person meetings [11]. Other online resources and technological tools for project management and collaboration, such as instant messaging applications, could contribute to the generation of knowledge and facilitate communication between research team members and collaborators [12].

## Rule 4: Comply with regulatory, ethical, and research integrity guidelines

A research team should have a strong commitment to comply with scientific integrity principles and best practices [13], highlighting the requirement for establishing internal strategies that promote the continuous adherence to national and local ethical regulations, such as guaranteeing the confidentiality of clinical data from participants [14]. In an era of electronic publication and social media [15], failures of scientific integrity or occurrence of research misconduct are even more visible.

In this context, in addition to international guidelines, countries have different types of laws and local regulations related to ethical and research integrity aspects, which should be actively taken into account by the research team. In terms of research involving human subjects, 2 main aspects emerge: approval by an institutional research ethics committee, which is commonly required by funders before the start of the project, and the signing of informed consent forms by participants [16], a process that should be carefully monitored. Regarding research with animal models, approval by an institutional animal ethics committee is commonly required [17]. In addition, other legal and administrative permissions, such as those from external or public institutions, might be needed in certain cases.

A Scientific Integrity Consortium, composed by representatives from 27 institutions from the United States and Canada, has developed a set of 9 core principles and best practices for scientific integrity that every researcher should comply with [18]. Some of these principles, of interest for teams carrying out research projects, are requiring universal training on responsible research practices, encouraging reproducibility of research and strengthening scientific integrity oversight [18].

## Rule 5: Follow predefined data analysis plans

An adequate application of key statistical concepts, such as the calculation of sample sizes, is important not only for the analysis of results, but also for design and execution of research projects [19,20]. In this context, data analysis plans are common key components of grant applications, predefining aspects such as definitions of groups, key variables to be analyzed, and statistical tests to be used [21]. Many funders require data management plans and a previous Ten Simple Rules paper gave advice about its creation [22]. Following those predefined plans would facilitate an adequate analysis of data [23], avoiding “p-hacking” [24], among other inadequate practices. In multiple research areas, there has been a growth in carrying out preregistration of studies [25,26] and recently Lakens has provided recommendations about when and how to deviate from preregistrations [27]. In the era of Open Science, a research team must be aware that scientific journals may require them to share their data plans when submitting a derived manuscript [28,29].

## Rule 6: Use validated methods for data collection and keep backups of data and analyses

The use of well-established methods for data collection, such as the employment of previously validated psychosocial scales [30] or well-known and reliable molecular methodologies, is key for obtaining high-quality research results [22]. A periodic monitoring of data quality is beneficial for research projects [31] and there are multiple approaches for doing so (such as the use of positive and negative controls or external standards, among others), depending on the specific methods used. As an example from molecular methods [32], a positive control is a sample known to have the feature of interest (such as a target for PCR amplification) and a negative control is a sample known as not having the feature of interest.

In some cases, the project might involve the creation, adaptation, or refinement of novel methods [30], which usually requires time and resources for their comparison with previous approaches. In many cases, an initial pilot phase [33] allows the identification of minor adjustments needed for data collection on a larger scale. In multicenter projects, it is advised that all participating sites employ the same protocols.

Misplacement, accidental damage, or loss of research data, such as results from phenotypic evaluations or molecular studies, would be catastrophic for any research project. In this context, strategies such as the use of Electronic Laboratory Notebooks [34,35], in addition to the frequent employment of multiple backups (in the cloud and in different computers) would avoid the loss of research data [22]. Constant backup of derivative files, with evolving versions of data analyses and manuscripts, is also recommended. An adequate structure of databases [36] involves their complete annotation and facilitates future data reanalysis. Another previous Ten Simple Rules article about digital data storage [29] would be a very useful resource for researchers. In addition, Boland and colleagues wrote an interesting paper about enabling multisite collaborations through data sharing [37].

## Rule 7: Implement the research budget and promote adequate administrative management

Although there are differences between calls for applications and between funders, there are 2 main types of costs, direct and indirect. Direct costs are related to the specific needs of the project and commonly include categories such as personnel, consultations and subcontracts, equipment, supplies, and travel, among others [12,38]. On the other hand, indirect costs are funds to cover the research infrastructure of the institution [39].

Commonly, costs associated with personnel are some of the largest in a research budget and there are previous suggestions regarding the adequate selection, recruiting, hiring, and management of scientific personnel [9].

There are some previous recommendations regarding the implementation of the research budget, such as the need for its revisions after the notice of award, the importance of including projections of inflation in multiyear grants and taking into account the possibility of having increased costs for certain categories [40]. Of particular interest for certain world regions, such as the Global South, there is the common need of considering the increased costs and times related to importing certain equipment and reagents from abroad. In terms of project management, which involves multiple administrative aspects, certain aspects are key, such as the need for strategic planning, adequate communication, and frequent monitoring, among others [8]. Additional elements to take into account are the constant need for training on budget management for the PIs, the adequate communication between the PI and the Project Manager, and having frequent administrative support from the institution [38].

## Rule 8: Assign enough time for reports

Final, and partial or progress, reports are major deliverables from research projects and their elaboration commonly involves a large amount of time and dedication. Partial or progress reports are quite useful for evaluating the performance of project activities in previous periods and adequately planning experiments and analyses for upcoming periods.

Final reports include a description and discussion of the results obtained and the perspectives for future studies, in addition to budget reports and generated deliverables. In many cases, it involves weeks of work and the participation of several researchers and support staff. Although its writing would need the involvement of all team members, the coordination of its elaboration is commonly a major responsibility of the PI (in close collaboration with the PM, when possible). As previously discussed, an adequate documentation of research procedures and findings would diminish the possibility that the departure of team members, among other unexpected events, negatively affect the elaboration of the research reports.

## Rule 9: Publish the findings and share the results of your project

Publication in peer-reviewed scientific journals remains one of the main forms to communicate research findings [41]. Publishing your positive or negative results, avoiding an overinterpretation of actual research findings [42], facilitates that the international scientific community receives and discusses the results and conclusions from your project [43]. In addition to original articles, which are the primary form of publication for new research results, consider other types of articles such as reviews, viewpoints, perspectives, and special articles to disseminate your insights about a research topic. A recent Ten Simple Rules paper provided suggestions for writing Registered Reports [44], which are a type of research publication where the proposed methodology is peer reviewed prior to data collection, to avoid publication and reporting biases. It involves 2 stages: Stage 1, where the introduction and proposed methods and analysis plans are reviewed, and Stage 2, where the results and discussion are included and reviewed [44].

Following international standards for the reporting of studies, such as those from the EQUATOR Network [45], promotes an adequate presentation of research findings. From an Open Science perspective, deposition of open research data in public repositories promotes transparency of results [46, 47] and facilitates replications of results and secondary analyses [48]. In this context, the FAIR Guiding Principles are important and involve the following aspects: being Findable, Accessible, Interoperable, and Reusable [49]. Many funding organizations have policies with specific requests about research data sharing and some examples of these policies can be found at: https://sharing.nih.gov/data-management-and-sharing-policy (for the NIH, USA) and https://wellcome.org/grant-funding/guidance/data-software-materials-management-and-sharing-policy (for the Wellcome Trust, UK).

## Rule 10: Share your results with the general public

Public outreach is another major aspect of scientific research [50], particularly because scientific projects are commonly taxpayer-funded, among others. In this regard, appropriate communication of research results to the public is of paramount importance, which involves strategies such as talks or texts oriented for the communities, using an easily understandable language [51] and avoiding exaggeration or misrepresentation of the actual research findings [52]. In addition, communication of research results at national and international conferences [53], and to other major stakeholders, such as professional societies or associations of patients, is also recommended. Social media, infographics, and podcasts are evolving as useful tools for the dissemination of new scholarly material and resources [53]. Consequently, it is becoming increasingly important for research teams to undergo training in the effective dissemination and knowledge translation of their work [54].

## Future considerations

Several of the Ten Simple Rules presented here are not exclusive to the process of carrying out funded research projects, as they are also necessary for other related scientific processes, such as the writing of grant applications or manuscripts. Scientific research is in constant evolution, and it is possible that in the near future, the execution of research projects also changes, taking into account aspects such as the increase of international mega-collaborations [55], the growth in the use of automated and high-throughput tools, including recent tools from generative artificial intelligence, and the constant need to verifying the integrity and quality of research findings [39], among others. Of particular importance, researchers should always carry out research projects with the highest standards of ethics and research integrity [56], avoiding negative practices, such as “Helicopter Research” [57], or gift or ghost authorship [10]. For the individual research teams, each new project is an opportunity to learn from both failures and successes, in order to refine and improve management strategies for future research initiatives. Finally, research institutions and groups should consider the need for frequent training activities related to multiple aspects of the execution of scientific projects [38].
